# Predisposition to apoptosis in keratin 8-null liver is related to inactivation of NF-κB and SAPKs but not decreased c-Flip

**DOI:** 10.1242/bio.20134606

**Published:** 2013-05-29

**Authors:** Jongeun Lee, Kwi-Hoon Jang, Hakhyun Kim, Younglan Lim, Sujin Kim, Han-Na Yoon, In Kwon Chung, Jürgen Roth, Nam-On Ku

**Affiliations:** Department of Integrated OMICS for Biomedical Science, WCU Program of Graduate School, Yonsei University, Seoul 120-749, Korea

**Keywords:** Keratin, Intermediate filaments, Liver, c-Flip, Apoptosis, NF-κB, SAPK

## Abstract

Keratin 8 and 18 (K8/K18) are major intermediate filament proteins of liver hepatocytes. They provide mechanical and nonmechanical stability, thereby protecting cells from stress. Hence, K8-null mice are highly sensitive to Fas-mediated liver cell apoptosis. However, the role of c-Flip protein in K8-null related susceptibility to liver injury is controversial. Here we analyzed c-Flip protein expression in various K8 or K18 null/mutant transgenic livers and show that they are similar in all analyzed transgenic livers and that previously reported c-Flip protein changes are due to antibody cross-reaction with mouse K18. Furthermore, analysis of various apoptosis- or cell survival-related proteins demonstrated that inhibition of phosphorylation of NF-κB and various stress activated protein kinases (SAPKs), such as p38 MAPK, p44/42 MAPK and JNK1/2, is related to the higher sensitivity of K8-null hepatocytes whose nuclear NF-κB is rapidly depleted through Fas-mediated apoptosis. Notably, we found that NF-κB and the studied protein kinases are associated with the K8/K18 complex and are released upon phosphorylation. Therefore, interaction of keratins with cell survival-related protein kinases and transcription factors is another important factor for hepatocyte survival.

## Introduction

Keratins are important cytoskeletal proteins and their expression is restricted to epithelial cells. Keratin monomers – one from type I (K9–K28, K31–K40) and the other from type II (K1–K8, K71–K86) – assemble into heteropolymers forming intermediate filaments (IF) (Eriksson et al., 2009; Herrmann et al., 2009; Kim and Coulombe, 2007; Moll et al., 2008; Schweizer et al., 2006). Keratins are tissue-specific cytoskeletal proteins and their mutations cause organ-specific human diseases (Coulombe et al., 2009; Omary et al., 2004; Omary et al., 2009). For example, K8 and K18 proteins are the major cytoskeletal proteins of epithelial cells in digestive organs such as liver. The K8/K18 expression in hepatocytes relates to liver-specific diseases observed in mutant K8 or K18 transgenic mouse models (Ku et al., 2007; Omary et al., 2009). These experimental findings led to human liver-disease related studies that identified K8/K18 variants in patients with liver diseases of multiple etiologies (Ku et al., 2007; Omary et al., 2009; Strnad et al., 2012). In addition, transgenic mice models showed the significance of K8/K18 proteins for liver protection against both mechanical and nonmechanical stress caused by toxic chemicals and apoptosis-inducing agents (Ku et al., 2007; Magin et al., 2007; Marceau et al., 2007).

The death receptor CD95 (Fas)-mediated apoptosis can be induced by Fas ligand (FasL) binding (Taylor et al., 2008; Wilson et al., 2009). In mouse liver, it can be induced by the administration of Fas antibodies (Ogasawara et al., 1993). This extrinsic pathway requires the recruitment of the Fas-associated protein with death domain (FADD) that binds to the intracellular region of the death receptor and forms the death-inducing signaling complex (DISC) along with procaspase-8. Autoproteolytic conversion of procaspase-8 into activated caspase-8 initiates a caspase cascade leading to apoptosis. Flice-like inhibitory protein (c-Flip) is a protein that takes part in the regulation of apoptosis by preventing caspase-8 activation at the DISC, thereby blocking the caspase cascade (Taylor et al., 2008; Wilson et al., 2009).

Mitogen-activated protein kinases (MAPKs) are critical signaling molecules to transmit extrinsic stimuli into diverse cellular processes including apoptosis, mitosis, cell motility, differentiation and development (Cuevas et al., 2007; Gallo and Johnson, 2002; Wagner and Nebreda, 2009). MAPKs are divided into three subfamilies, namely JNK1/2, p38 MAPK and p44/42 MAPKs (ERK1/2). Their substrates are transcription factors, membrane receptors and other downstream protein kinases that become activated and are linked to the regulation of gene expression under various stimuli. For example, activation of transcription factors (e.g., p53, NF-κB, c-Jun, ATF2), and protein kinases (e.g., RSKs, MKs, MSKs) is directly or indirectly associated with MAPK signaling pathways (Avruch, 2007; Wagner and Nebreda, 2009). The variety of their substrates confers broad impact on gene regulation, which is ultimately under the control of MAPKs. Notably, all three subfamilies of MAPKs bind K8/K18 and phosphorylate K8 (Gilbert et al., 2004; He et al., 2002; Ku et al., 2002). The abundance of cytoplasmic keratins provides plentiful binding capacity for MAPKs, whereas keratin ablation or mutations that are no more MAPK substrates may lead to increased MAPK availability to other molecules that modulate cell function.

Indeed, K8-null mice are highly susceptible to Fas-mediated liver injury (Gilbert et al., 2001). The increased susceptibility in K8-null liver was reported to be due to a drastic reduction of c-Flip protein and associated with inhibition of phosphorylation/activation of p44/42 MAPKs (Gilbert et al., 2004). On the contrary, our previous studies showed no change of mouse c-Flip protein in K8 or K18 ablation/mutation transgenic livers (Ku and Omary, 2006). This discrepancy may have resulted from the use of two different c-Flip antibodies, namely antibody (Ab) # 06-864 (Gilbert et al., 2004) and Ab # 06-697 (Ku and Omary, 2006). Here, we compare levels of mouse c-Flip protein expression in various mutant K8 or K18 transgenic mice liver. Our data show that the c-Flip protein levels remain unchanged in the keratin mutant transgenic livers including K8-null liver. The band detected by Ab # 06-864 and proposed to be mouse c-Flip protein (Gilbert et al., 2004), was found to be due to cross-reactivity of this antibody with mouse K18 protein, which is drastically reduced in K8-null liver and therefore excludes c-Flip as a pathogenic factor. Indeed, our results show that the higher sensitivity of K8-null hepatocytes to Fas-mediated apoptosis is due to inhibition of phosphorylation/activation of NF-κB and SAPKs, which results in depletion of nuclear NF-κB and leads to a rapid initiation of massive apoptosis.

## Results

### Ab # 06-864 against c-Flip cross-reacts with mouse K18

**Fig. 1.**
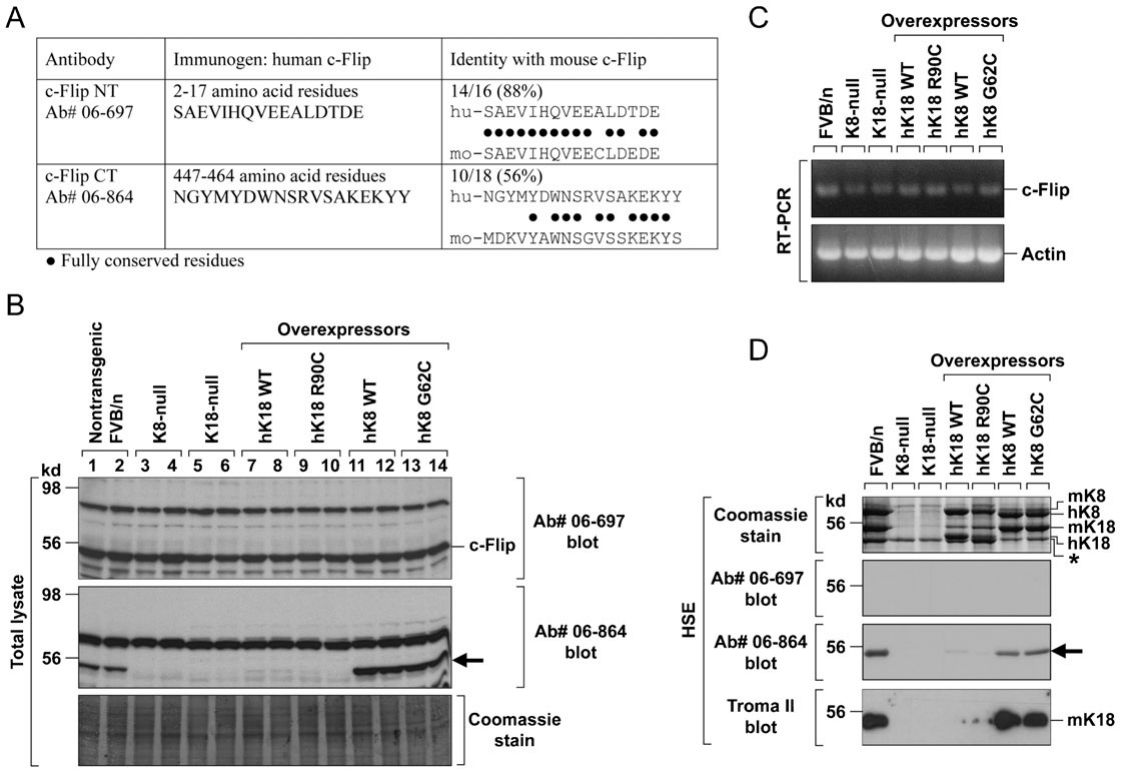
Ab # 06-864 cross-reacts with mouse K18. (**A**) Alignment of two human c-Flip immunogen sequences with the corresponding mouse c-Flip. The immunogen identity to mouse c-Flip is 88% for Ab # 06-697 and 56% for Ab # 06-864. (**B**) Total liver homogenates from the indicated transgenic mouse strains were resolved in SDS-PAGE followed by Coomassie Blue staining to verify an equal amount of protein per lane or immunoblotting with indicated antibodies. Immunoblots with Ab # 06-697 showed a ∼55 kd band in all analyzed livers regardless of K8/K18 absence or mutation (this is a full size immunoblot instead of a single panel cut as published previously by Ku and Omary in their figure 3D (Ku and Omary, 2006)). In contrast, immunoblots with Ab # 06-864 showed no ∼55 kd band (arrow) in livers of K8-null, K18-null, hK18 WT, and hK18 R90C mice. (**C**) Total RNA was prepared from livers and amounts of mouse c-Flip mRNA were determined by RT-PCR with primer sets as described in Materials and Methods. (**D**) A keratin enriched fraction, by high salt extraction (Ku et al., 2004), was prepared from livers of nontransgenic FVB/n mice, K8- or K18-null mice, or transgenic mice that overexpress human wild-type or mutant K8/K18. Coomassie Blue staining shows K8/K18 bands in different transgenic livers, except for K8-null or K18-null livers due to rapid degradation in the absence of its heterodimeric partner. Mouse K8/K18 and human K8/K18 can be distinguished by their different molecular mass. Immunoblot of high salt extracts with Ab # 06-697 showed no band, whereas Ab # 06-864 produced ∼55 kd bands (arrow) at the position of mouse K18. This was verified by immunoblot using a mouse K18 specific antibody, termed Troma II (lower panel). Asterisk indicates unidentified band.

A previous study showed dramatic decrease of mouse c-Flip in K8-null hepatocytes (Gilbert et al., 2004) using Ab # 06-864. In contrast, we reported that the level of c-Flip protein was similar in mouse liver irrespective of the genotype, including K8-null liver, by using a different c-Flip antibody, namely Ab # 06-697 (Ku and Omary, 2006). Of note, different immunogens were used to raise these antibodies (Fig. 1A). The immunogen for Ab # 06-864 was a peptide corresponding to amino acid residues 447–464 of human c-Flip. When we compared the 18 amino acids of the human c-Flip sequence with the corresponding mouse c-Flip, 10 matches were found which corresponded to 56% identity (Fig. 1A). The immunogen for Ab # 06-697 was a peptide corresponding to amino acid residues 2–17 of human c-Flip. Comparison between the amino acid sequence of this immunogen and mouse c-Flip showed 14 conserved residues out of the 16, which corresponded to 88% identity (Fig. 1A). In Fig. 1B, immunoblots using the two different antibodies show striking differences in the protein band pattern in transgenic as compared to nontransgenic mouse liver. Immunoblot with Ab # 06-697 showed a ∼55 kd band regardless of the keratin mutation in the different transgenic mice (Fig. 1B). In agreement with this, we observed similar amounts of c-Flip mRNA in the analyzed mice livers by RT-PCR (Fig. 1C). However, in immunoblot using Ab # 06-864, the ∼55 kd band was undetectable not only in K8-null, which confirms the results of the previous study of Gilbert et al. (Gilbert et al., 2004), but was also undetectable in K18-null mice, hK18 WT and hK18 R90C overexpressing mice (Fig. 1B). The obvious discrepancy between the expression of mouse c-Flip mRNA and c-Flip protein points to differences in the specificity of two used antibodies. We reasoned that the Ab # 06-864 might cross-react with mouse K18 for the following reasons. First, both mouse K18 and mouse c-Flip have a molecular mass of ∼55 kd. Second, a common feature of the four different transgenic livers (K8 or K18-null and hK18 WT/mutant transgenic livers) in which no ∼55 kd band was detected by Ab # 06-864 (Fig. 1B), was the absence or dramatic reduction of mouse K18 (Ku and Omary, 2006).

To verify the cross-reactivity of Ab # 06-864 with mouse K18, high salt extracts (HSE) from livers of various transgenic mice were prepared to obtain the total liver keratin pool. Coomassie blue staining of the HSE showed no mouse K18 in K8-null/K18-null livers and dramatically reduced mouse K18 in hK18 WT/R90C overexpressing livers (Fig. 1D, upper panel). Overexpression of hK8 WT and hK8 G62C in transgenic liver did not affect the level of mouse K18 although it caused reduction in the amount of mouse K8 protein. Immunoblots with Ab # 06-697 showed no bands in the HSE samples (Fig. 1D, 2nd panel), whereas Ab # 06-864 produced ∼55 kd bands in samples from nontransgenic mice, hK8 WT and hK18 G62C overexpressing mice (Fig. 1D, 3rd panel). The bands overlapped with the position of mouse K18 protein in those mice (Fig. 1D, 4th panel). These results demonstrate that Ab # 06-864 is cross-reactive with mouse K18.

### Effect of K8 absence on apotosis-associated cell signaling

Since we have shown that the Ab # 06-864 cross-reacts with mouse K18 and thus is not specific for mouse c-Flip, the proposed role of c-Flip in the pathogenesis of Fas-induced liver damage seems not to be justified. This is also supported by our lethality studies on hK18 WT mice. Like in K8-null liver, no band was detected by Ab # 06-864 in hK18 WT liver, in contrast to nontransgenic FVB/n liver (Fig. 1B). However, the lethality percentage of nontransgenic FVB/n mice and of hK18 WT mice caused by different forms of stress, i.e. administration of Fas antibody alone, streptozotosin or Fas antibody combined with PUGNAc (an inhibitor of N-acetyl-D-glucosaminidase) (Ku et al., 2010), was similar (Table 1).

**Table 1.**
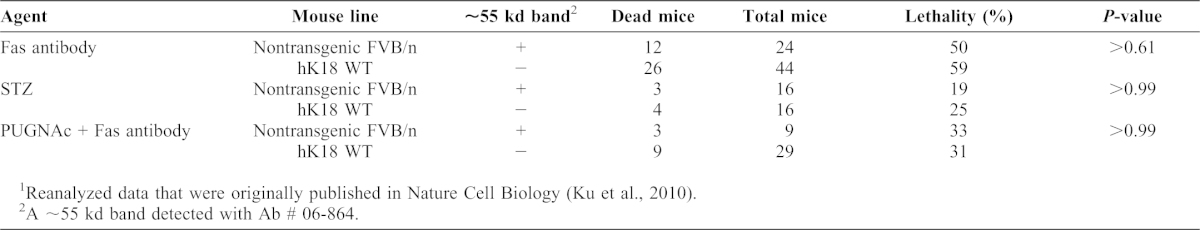
Mouse lethality after injection of different agents^1^. Two groups of mice [one nontransgenic FVB/n (∼55 kd band positive for Ab #06-864) and the other hK18 WT overexpressed transgenic mice (negative for Ab #06-864)] were examined for their lethality under three different stressed conditions. *P*-value in each case is >0.05, thereby confirming no significant difference in mouse lethality between the two groups due to what was proposed to be a ‘c-Flip’.

**Fig. 2.**
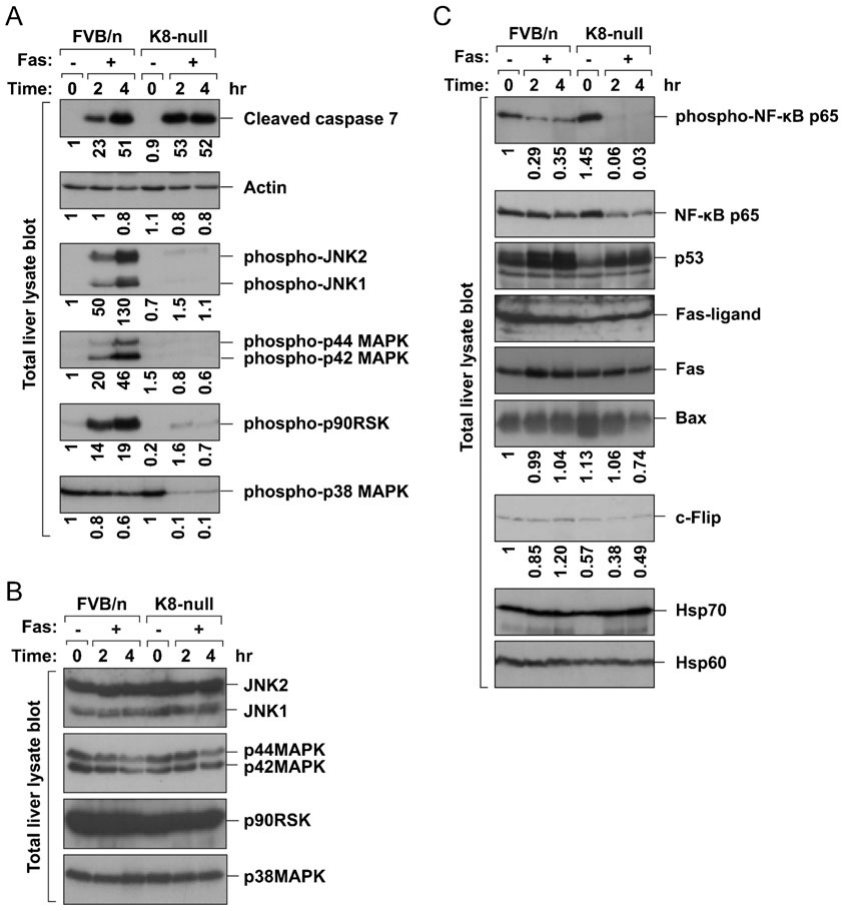
K8 ablation inhibits activation of SAPKs and NF-κB. Nontransgenic FVB/n or K8-null mice were injected intraperitoneally with Fas Ab (0.15 mg/kg body weight) to induce liver apopotosis. After 2 and 4 hrs, liver homogenates were prepared and immunoblotted with antibodies against cleaved caspase 7 for apoptotic level and phospho-SAPKs for SAPK activation (**A**), nonphospho-SAPKs for SAPK protein level (**B**), and additional cell survival/apoptosis-associated proteins including NF-κB and p53 transcription factors (**C**). Note that phosphorylation/activation of SAPKs and NF-κB was dramatically inhibited in Fas-treated K8-null liver as compared with nontransgenic FVB/n mice. In addition, inhibited phosphorylation of p90RSK (in panel A), a substrate of p44/42 MAPK, is likely caused by inactive p44/42 MAPK in Fas-treated K8-null liver. Numbers below immunoblots represent the relative pixel intensity of each band.

In terms of increased susceptibility of K8-null mice after Fas administration shown in previous study (Gilbert et al., 2001), we observed the same pattern of results that showed the increased mortality and liver damage in K8-null mice after Fas treatment (supplementary material Fig. S1). To gain further insight in the molecular mechanism of Fas-induced liver damage in K8-null mice, we analyzed the expression of several apoptosis-related proteins and the phosphorylation/activation of protein kinases and transcription factors, all of which are known to be associated with cell survival or apoptosis. Nontransgenic FVB/n mice and K8-null mice received Fas antibody intraperitoneally to induce liver apoptosis and liver lysates were analyzed by immunoblotting. Increased apoptosis in K8-null livers as compared to nontransgenic FVB/n mice was observed by increased formation of cleaved caspase 7 fragment (Fig. 2A). Furthermore, phosphorylation/activation of p38 MAPK, p44/42 MAPK, JNK1/2 and p90RSK, all regulators of cell-cycle progression and survival (Anjum and Blenis, 2008; Wagner and Nebreda, 2009), was dramatically inhibited in K8-null livers as compared to nontransgenic FVB/n mice under Fas-mediated apoptosis (Fig. 2A). Inhibition of phosphorylation of p44/42 MAPK in Fas-treated K8-null liver prevents subsequent phosphorylation of ribosomal S6 kinase (p90RSK) (Fig. 2A), known as a substrate of p44/42 MAPK (Anjum and Blenis, 2008). Notably, the protein amount of all studied kinases was similar in immunoblots of both nontransgenic FVB/n and K8-null livers (Fig. 2B). Consistent with these *in vivo* findings, a dramatic inhibition of p44/42 MAPK phosphorylation in *ex vivo* cultured K8-null hepatocytes was observed in a previous study (Gilbert et al., 2004).

Next, we tested the effect of K8 ablation on phosphorylation/activation of transcription factors and the expression of several apoptosis-related proteins. Remarkably, phosphorylation of NF-kB p65 was blocked in Fas treated K8-null livers and the expression of NF-kB target genes, such as Bax (Grimm et al., 2005) and c-Flip (Kreuz et al., 2001), was downregulated in the K8-null livers (Fig. 2C). Although the c-Flip band of K8-null (lane 4 in Fig. 2C) was weaker than that of FVB/n under basal conditions (lane 1 in Fig. 2C), it is likely due to the variation of c-Flip expression in individual mouse, which is independent of K8 expression. The densitometric quantification of c-Flip expression from 3 mice/strain showed the c-Flip expression in both mice strains was similar under basal conditions (supplementary material Fig. S2). On the other hand, p53 expression was similar in livers of both mice strains independent of Fas treatment (Fig. 2C). The phosphorylation of p53 could not be analyzed for technical reason. We observed no differences in other apoptosis-associated proteins and in stress-associated proteins such as Hsp70/Hsp60 in livers of both nontransgenic FVB/n and K8-null livers independent of Fas treatment (Fig. 2C).

Taken together, predisposition to apoptosis in K8-null liver is related to the lower level of phosphorylated kinases/NF-κB p65. The lower level is not likely due to rapid degradation of the proteins resulted from a consequence of faster apoptosis in K8-null livers since the amounts of each kinase (Fig. 2B) and NF-κB p65 (Fig. 2C) are similar in nontransgenic FVB/n liver and K8-null liver. In addition, the levels of cleaved caspase 7 in FVB/n and K8-null livers after 4 hr treatment of Fas antibody are similar, but the phosphorylation of the kinases/NF-κB p65 is dramatically inhibited in the K8-null liver (Fig. 2A) whereas the amount of the proteins are similar in both livers (Fig. 2B). Hence, it is likely that K8 is involved in phosphorylation/activation of the proteins by an unknown mechanism.

### Interaction between K8/K18 and protein kinases/transcription factors

**Fig. 3.**
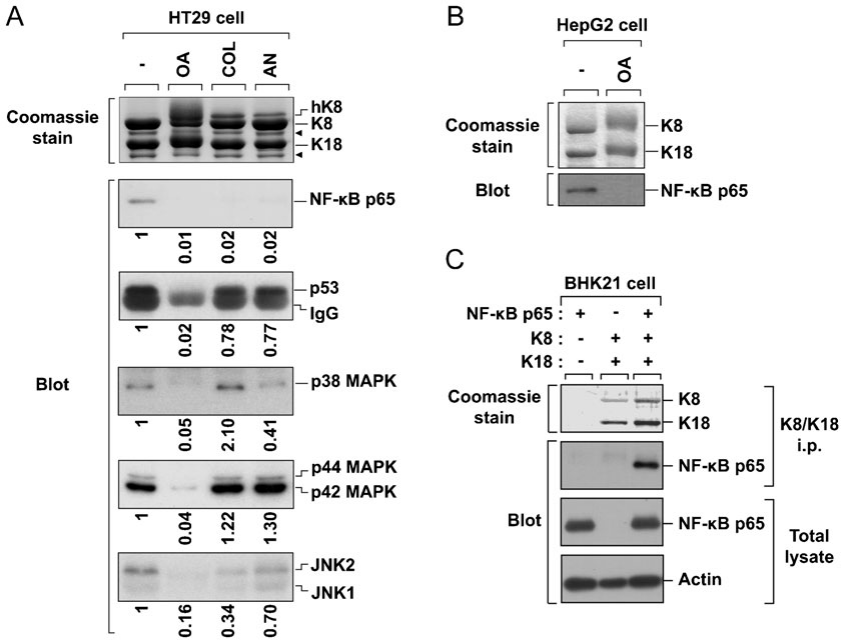
K8/K18 associates with SAPKs and transcription factors NF-κB and p53. (**A**) HT29 (human colon carcinoma) cells were incubated in the presence or absence of okadaic acid (OA) for 2 hrs, colcemid (Col) for 36 hrs, or anisomycin (An) for 8 hrs. K8/K18 immunoprecipitates were obtained and resolved by 10% SDS-PAGE. Duplicate gels were stained with Coomassie Blue or transferred to PVDF membrane and immunoblotted with the indicated antibodies. Arrowheads indicate degraded K8 or K18. Note that NF-κB/p53 and SAPKs are associated with K8/K18 under basal conditions and released from the keratin complexes upon phosphorylation. (**B**) HepG2 (human hepatocellular carcinoma) cells were incubated in the presence or absence of OA and analyzed as described in panel A. As shown in HT29 cells, NF-κB p65 in HepG2 cells interacts with K8/K18 under basal conditions and dissociates from them upon phosphorylation. (**C**) BHK21 cells were transfected only K8/K18 or K8/K18 with NF-κB p65. K8/K18 immunoprecipitates and total lysates were prepared and immunoblotted with indicated antibodies.

Given that the enhanced susceptibility to liver injury in K8-null liver is associated with a dramatic reduction in the level of phosphorylation/activation of protein kinases and NF-κB p65, we examined whether they interact with K8/K18. We used the HT29 colon carcinoma cell line, which expresses high level of endogenous K8/K18. The following conditions are tested: treatment with okadaic acid (OA, a phosphatase inhibitor), colcemid (Col, an antimitotic agent), and anisomycin (An, an apoptosis inducer). Strikingly, we observed an interaction between NF-κB p65 and K8/K18 under basal conditions, and the dissociation of the complexes under the various stress conditions including OA treatment (Fig. 3A). We also detected the dissociation of the complexes in the HepG2 hepatocellular carcinoma cell line after OA treatment, as found in HT29 cells (Fig. 3B). These results demonstrated that in both cell lines NF-κB p65 was released from the K8/K18 complex in a phosphorylation-dependent manner. In addition, NF-κB p65 associated with K8/K18 was observed in BHK21 cells overexpressing NF-κB p65 and K8/K18 (Fig. 3C). In the other hand, under anisomycin-induced apoptosis, NF-κB p65–keratin interaction was disrupted whereas p53–keratin interaction remained almost unaffected (Fig. 3A). Hence, it is likely that NF-κB p65, rather than p53, may be a critical factor to be regulated by keratin complexes under apoptosis. Furthermore, interaction of p38, p44/42 and JNK1/2 to K8/K18 was also observed under basal conditions and remained almost unaffected by Col or An treatment (Fig. 3A). However, similar to the case of the transcription factors, OA treatment resulted in the dissociation of the complexes, implying the inhibition of their interactions by protein phosphorylation (Fig. 3A). Taken together, our findings indicate that the binding of keratins with cell survival-related protein kinases and transcription factors is important for cell survival and is pathogenic factor in liver injury.

### Effect of K8 absence on localization of NF-κB p65

**Fig. 4.**
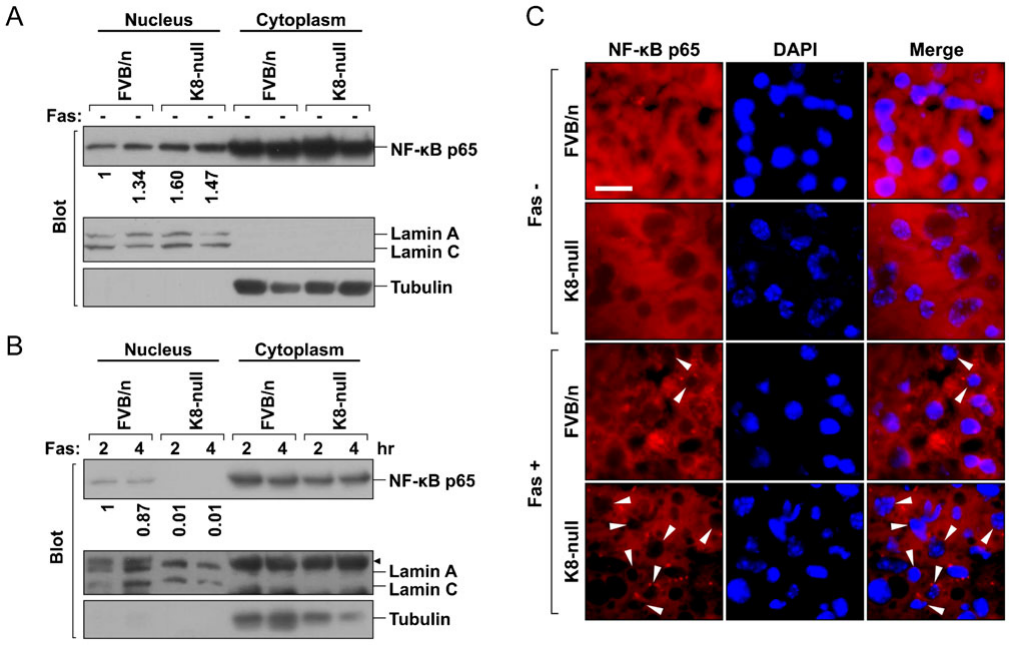
Nuclear NF-κB p65 in K8-null hepatocytes is rapidly depleted following liver injury. (**A**) Hepatic nuclear and cytoplasmic fractions were prepared from nontransgenic FVB/n and K8-null mice under basal conditions and then were analyzed by immunoblotting. Each lane represents the analysis of one individual mouse liver. The subcellular distribution of NF-κB was similar in both control and K8-null hepatocytes under basal conditions. Nuclear lamin A/C and cytoplasmic tubulin were used to demonstrate the purity of cell fractions. (**B**) Immunoblot analysis as in panel A of nontransgenic FVB/n and K8-null mice livers after Fas administration. Note the absence of NF-κB p65 in the nuclear fraction of K8-null hepatocytes upon Fas treatment. Arrowhead indicates nonspecific bands. (**C**) Immunofluorescence for NF-κB p65 (red) in liver sections stained with DAPI (blue) to visualize nuclei. Livers from PBS- or Fas-treated mice for 2 hrs were isolated and analyzed by immunostaining. Note the loss of immunofluorescence for nuclear NF-κB p65 in most K8-null hepatocytes after Fas treatment. Arrowheads point to NF-kB p65 depleted nuclei. Scale bar: 10 µm.

Cytoskeletal proteins, such as F-actin, tubulin and vimentin, are involved in the transport and/or localization of p53 (O'Brate and Giannakakou, 2003). Since it is established that the function of NF-κB p65 is modulated by its subcellular localization (Meylan et al., 2009), we compared its distribution in nontransgenic FVB/n and K8-null liver. First, nuclear and cytosolic fractions were analyzed by immunobloting. Localization of NF-κB p65 was similar in livers of both nontransgenic FVB/n and K8-null animals under basal conditions (Fig. 4A). However, NF-κB p65 nuclear retention was markedly diminished in K8-null liver under Fas treatment as compared with control liver (Fig. 4B). Next, we examined the cellular distribution of NF-κB p65 by immunofluorescence. Consistent with the immunoblot results, NF-κB p65 nuclear depletion rapidly occurred in Fas treated K8-null liver. Most nuclei of K8-null hepatocytes lacked nuclear NF-κB p65 immunofluorescence at 2 hr after Fas treatment, whereas ∼15% nuclei of nontransgenic FVB/n hepatocytes lacked NF-κB p65 (Fig. 4C). Hence, in the case of increased susceptibility of K8-null mice to liver injury, a rapid depletion of nuclear NF-kB p65 (Fig. 4) together with dephosphorylation/inactivation of NF-kB p65 (Fig. 2C) occurred, and thereby blocked NF-κB p65 mediated pro-survival pathway, which leading to facilitate apoptosis under liver injury.

## Discussion

**Fig. 5.**
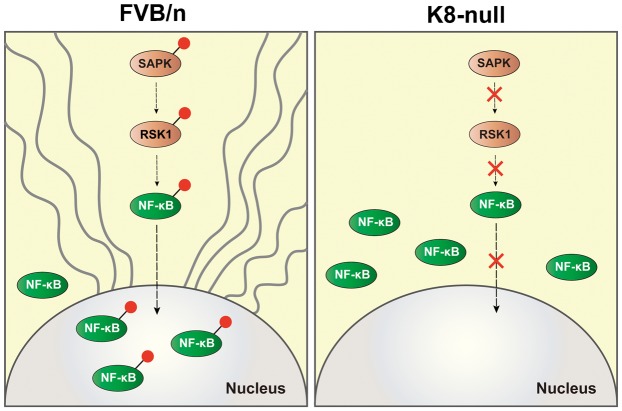
Model depicting different patterns of SAPKs activation and NF-κB localization depending on the presence or absence of keratin filament under stress. Following Fas-mediated liver injury, SAPKs such as p44/42 MAPK and JNK1/2 are phosphorylated/activated in control FVB/n liver but not in K8-null liver, which indicates K8 involvement in the activation of SAPKs by an unknown mechanism. In the control liver, the activated SAPKs phosphorylate downstream kinases such as RSK1 that can finally phosphorylate NF-κB. This promotes NF-κB movement into the nucleus where it may initiate pro-survival signaling pathways, thereby delaying liver damage. On the other hand, the unphosphorylated SAPKs in K8-null livers cannot activate downstream kinases such as RSK1 and as a consequence NF-κB cannot be phosphorylated either. This hinders its nuclear localization, interrupts the initiation of the NF-κB-mediated pro-survival pathways and eventually leads to a rapid progression of apoptosis. Grey waved line and red dot denote keratin filaments and phosphorylation, respectively.

The present study was prompted by discrepant findings of the importance of c-Flip for the enhanced susceptibility of K8-null mice towards liver-injury causing drugs (Gilbert et al., 2004; Ku and Omary, 2006). The main findings of our study are as follows. An antibody specific for c-Flip detects no decrease in c-Flip levels in liver of K8-null and other keratin transgenic mice. Thus, c-Flip seems not to be a pathogenic factor for the enhanced susceptibility of K8-null mice for Fas-mediated apoptosis. Rather, we find that inactivation of NF-κB and various SAPKs, together with a rapid depletion of nuclear NF-κB, is an important pathogenic factor (Fig. 5).

Stress activated protein kinases, such as p44/42 MAPKs, p38 MAPK and JNK1/2, convert a variety of environmental stress signals into diverse cellular responses (Avruch, 2007). p44/42 MAPKs are phosphorylated/activated by mitogens and are upregulated in human cancers, whereas p38 MAPK and JNK1/2 activation results either in apoptosis or in pro-survival depending on cell type and intensity of stress (Wagner and Nebreda, 2009). Our data demonstrate inhibition of phosphorylation of all these protein kinases in K8-null liver under conditions of Fas-induced apoptosis. This implies that p38/JNK-mediated apoptosis may not be involved in increased lethality of challenged K8-null mice. Rather, SAPK-mediated pro-survival pathways may be blocked in K8-null liver under Fas-induced apoptosis. In agreement, inhibited phosphorylation/activation of NF-κB and deficiency in nuclear localization were observed in Fas-treated K8-null liver, despite constant p53 protein level. Interestingly, all these kinases were found to be associated with K8/K18 complexes under basal conditions, but were released in a phosphorylation-dependent manner. The biological significance of this interactions remains to be further investigated.

Another major finding in this work is the interaction of K8/K18 with the transcription factors p53 and NF-κB, which are apoptotic and pro-survival factors, respectively. Although the functional implications of this complex formation remain to be further determined, a biological significance of p53 binding to other cytoskeletal proteins such as vimentin, F-actin and tubulin was demonstrated in previous studies (O'Brate and Giannakakou, 2003). It has been shown that p53 is transported along microtubules to the nucleus and that this is important for p53 accumulation in the nucleus after DNA damage (Giannakakou et al., 2000). On the other hand, p53 accumulation in the cytoplasm of glioma cells depends on presence of vimentin, while nuclear accumulation is observed in vimentin-negative glioma cells (Klotzsche et al., 1998). An aberrant cytoplasmic sequestration of p53, therefore unable to act as a transcription factor, was found in various cancers including undifferentiated neuroblastoma (Moll et al., 1995) and hepatocellular carcinoma (Ueda et al., 1995).

NF-κB activation is involved in pro-survival pathways, leading to cancer development (Hayden and Ghosh, 2012; Perkins, 2012). However, little is known regarding the functional significance of cytoskeletal proteins in these signaling pathways. Although the molecular mechanism is not fully understood, the involvement of cytoskeletal proteins in the NF-κB signaling was previously reported. TNF-α mediated NF-κB activation is reduced by colchicine-mediated inhibition of microtubule formation, while taxol-mediated microtubule stabilization (even in the absence of TNF-α) enhances NF-κB activation (Jackman et al., 2009). Similarly, NF-κB phosphorylation/activation is interrupted in K8-null liver under stress as compared with control liver containing intact keratins (Fig. 2C). Together, this suggests that intact microtubules and keratins are important for the NF-κB signaling pathway.

NF-κB function is modulated by its subcellular localization. Under basal conditions, NF-κB p65/p50, which has a nuclear localization signal (NLS) is bound to IκB which has a nuclear export signal (NES). Thus, the complexes continuously shuttle between the nucleus and the cytoplasm although most of them are in the cytoplasm under steady state (Hayden and Ghosh, 2008). Previous studies on cell cultures showed that upon stimuli, IκB degradation and p65 phosphorylation occur, which results in nuclear translocation of NF-κB (Chen and Greene, 2004). However, our *in vivo* studies in livers demonstrate that: (i) NF-κB p65 phosphorylation, at S534 in the mouse protein, can be detected under basal conditions in both nontransgenic FVB/n and K8-null livers (Fig. 2C), (ii) NF-κB p65 phosphorylation is diminished with the occurrence of Fas-mediated apoptosis (Fig. 2C), and (iii) a rapid depletion of nuclear NF-κB occurs in highly susceptible K8-null hepatocytes following liver injury (Fig. 4). The loss of nuclear NF-κB is probably due to failure of NF-κB translocation in the nucleus and may be related to the inactivation of stress-activated protein kinases (SAPKs), such as MAPKs. Various studies of the noncanonical pathway have shown that NF-κB p65 is phosphorylated at S276 by RSK1 (Bohuslav et al., 2004) and at S536 by MSK1 (Chen and Greene, 2004; Vermeulen et al., 2003). Both kinases are downstream kinases regulated by MAPKs, such as p44/42MAPK and p38MAPK (Cargnello and Roux, 2011). Hence, it is likely that inactivation of SAPKs in K8-null hepatocytes under conditions of liver injury results in a deficient phosphorylation state of NF-κB. As a consequence, this hinders its nuclear localization, interrupts the initiation of the NF-κB-mediated pro-survival pathway and eventually leads to a rapid progression of apoptosis. A major challenge ahead is to address how keratins modulate NF-κB or p53 signaling. Future studies should aim to identify the interaction sites between keratins and the protein kinases/transcription factors and to reveal the relation between the intracellular distribution of the transcription factors and specific keratins by analyzing transgenic tissues. This may result in information about a possible survival advantage for hepatocytes provided by intact keratins.

## Materials and Methods

### Reagents and keratin-mutant mice

The following antibodies were used: c-Flip antibodies (Ab # 06-697 and Ab # 06-864, Upstate Biotechnology/Millipore); Fas antibody (for mouse injection; BD Biosciences); phospho- or nonphospho-p38, p90RSK, phospho-p90RSK (human T359/S363, mouse T348/S352), phospho-NF-κB p65 (human S536, mouse S534), phospho-p42, c-Jun N-terminal protein kinase (JNK) and c-Jun (Cell Signaling); Fas antibody (for immunoblotting), FADD and Bax (Upstate Biotechnology/Millipore); Fas-ligand (Santa Cruz Biotechnology). K8-null and hK18 WT mice were provided by Robert Oshima (The Burnham Institute, La Jolla, CA), K18-null mice by Thomas Magin (University of Leipzig, Leipzig, Germany) and hK8 WT/G62C and hK18 R90C mice were generated as described previously (Ku et al., 1995; Ku and Omary, 2006).

### Animal studies

All animal experiments were performed in accordance with the Korean Food and Drug Administration guidelines. Protocols were reviewed and approved by the Institutional Animal Care and Use Committee of the Yonsei Laboratory Animal Research Center. All mice were maintained in a specific pathogen-free facility. For the lethality experiments, mice were injected intraperitoneally as previously described (Ku et al., 2010): Fas Ab (0.15 mg/kg body weight), STZ (200 mg/kg), or PUGNAc (7 mg/kg) and received a second Fas Ab injection after 48 hrs. For the biochemical analyses, mice were treated with Fas Ab and livers were isolated after 2 hrs and 4 hrs. The livers were homogenized in phosphate-buffered saline containing 5 mM EDTA and a protease inhibitor cocktail (25 µg/ml aprotinin, 10 µM leupeptin, 10 µM pepstatin and 0.1 mM phenylmethyl-sulfonyl fluoride). Homogenized samples were used for preparation of total lysates or high salt extracts as described previously (Ku et al., 2004).

### Cell subfractionation

Homogenized liver samples were solubilized with lysis buffer A (0.1% CA, 0.01 M NaCl, 20% glycerol in PSB with 5 mM EDTA) and incubated on ice for 10 min. The cytoplasmic fraction was recovered after centrifugation (400 g, 5 min, at 4°C). The remaining pellet was washed three times with the lysis buffer A and was solubilized in lysis buffer B (0.1% CA, 0.5 M NaCl, 20% glycerol in PBS with 5 mM EDTA) followed by incubation on ice for 30 min and centrifugation (15,000 g, 5 min, at 4°C) to obtain the nuclear fraction. To verify the purity of the fractions, lamin A/C or tubulin were used as nuclear and cytoplasmic marker, respectively.

### Cell culture and keratin immunoprecipitation

HT29 (human colon carcinoma) cells were cultured in RPMI 1640 medium supplemented with 10% fetal calf serum and treated with okadaic acid (1 µg/ml for 2 hrs), colcemid (0.5 µg/ml for 36 hrs), or anisomycin (10 µg/ml for 8 hrs). HepG2 (human hepatocellular carcinoma) cells were incubated in EMEM (GIBCO) with 10% fetal calf serum and analyzed after treatment with okadaic acid (1 µg/ml for 2 hrs). K8/K18 immunoprecipitation was carried out using the mouse monoclonal antibody L2A1 (Ku et al., 2004).

### Biochemical analysis

Liver total lysates, high salt extracts or K8/K18 immunoprecipitates were resolved by 10% SDS-PAGE and then followed by either Coomassie Blue staining or transfer to PVDF membranes for immunoblotting. Immunoreactive proteins were visualized by enhanced chemiluminescence.

### Immunohistochemistry

Cryostate sections of mouse livers (control and Fas treated were fixed with acetone for 10 min at −20°C and air-dried for 1 hour. After three rinses with PBS, sections were blocked with 2.5% BSA in PBS (w/v) for 10 min. Afterwards, sections were incubated with NF-κB p65 antibody (Cell Signaling) diluted in PBS containing 15% goat serum and 2.5% BSA for 30 min. After rinses with buffer, sections were incubated with Texas-red labeled goat anti-rabbit IgG (Molecular Probes) for 30 min. Finally, the sections were stained with DAPI (Sigma–Aldrich) for 5 min and observed under Zeiss Axio Imager M2 microscope using a 100× objective.

### RT-PCR

Total RNA was isolated from mouse liver by using RNA extraction kit with easy-BLUE ™ (iNtRON) and cDNA synthesis was performed by Transcriptor First Strand cDNA Synthesis Kit (Roche) according to manufacturer's instructions. RT-PCR for mouse c-Flip and mouse β-actin was done with following primer sets as described previously (Gilbert et al., 2004): mouse c-Flip, 5′-AATGTGGACTCTAAGCCCCTGCAACC-3′ and 5′-CGTAGGAGCCAGGATGAGTTTCTTCC-3′; mouse β-actin, 5′-GTGGGCCGCCCTAGGCACCAG-3′ and 5′-CTCTTTGATGTCACGCACGATTTC-3′.

## Supplementary Material

Supplementary Material
