# Supplementary material for: Predisposition to apoptosis in keratin 8-null liver is related to inactivation of NF-κB and SAPKs but not decreased c-Flip
Source: Biol Open. 2013 May 29;2(7):695–702. doi: 10.1242/bio.20134606 (PMC3711037; doi:10.1242/bio.20134606)
Supplement: Supplementary Material [file supp_2_7_695__index.html]

Predisposition to apoptosis in keratin 8-null liver is related to inactivation of NF-κB and SAPKs but not decreased c-Flip — Predisposition to apoptosis in keratin 8-null liver is related to inactivation of NF-κB and SAPKs but not decreased c-Flip — Supplementary Material 

# Predisposition to apoptosis in keratin 8-null liver is related to inactivation of NF-κB and SAPKs but not decreased c-Flip

## bio.20134606 Supplementary Material

**Files in this Data Supplement:**

- Supplementary Material - Jongeun Lee et al. doi: 10.1242/bio.20134606
