# Supplementary material for: Predisposition to apoptosis in keratin 8-null liver is related to inactivation of NF-κB and SAPKs but not decreased c-Flip
Source: Biol Open. 2013 May 29;2(7):695–702. doi: 10.1242/bio.20134606 (PMC3711037; doi:10.1242/bio.20134606)
Supplement: Supplementary Material [file supp_bio.20134606_bio.20134606-s1.pdf]

Biology Open

Supplementary Material

Jongeun Lee et al. doi: 10.1242/bio.20134606

A

Mouse line	Dead mice	Total mice	Lethality %
FVB/n†	4	9	44
K8-null	7	9	78

† Nontransgenic mice

B

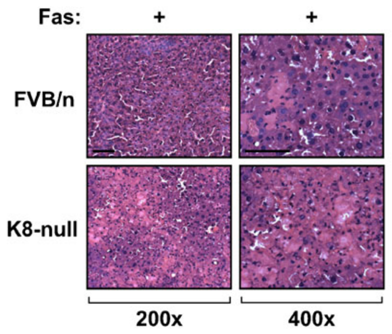

Fig. S1. Increased susceptibility of K8-null mice after Fas treatment. Nontransgenic FVB/n or K8-null mice were treated with Fas Ab (0.15 mg/kg body weight) to induce liver injury. (**A**) Mouse mortality was shown. (**B**) Histologic analysis. The livers were isolated after 3 hrs, fixed with 10% formalin and stained with hematoxylin/eosin. Note more severe liver damage in K8-null liver as compared with nontransgenic FVB/n liver. Scale bars: 50 µm.

A

	Lane	Mouse line	Area	Percent	Relative density
c-Flip in Fig. 1B	1	FVB/n	18098.421	21.859	1.00
	2	FVB/n	21229.158	25.64	1.17
	3	K8-null	21062.016	25.438	1.16
	4	K8-null	22406.572	27.062	1.24
c-Flip in Fig. 2C	1	FVB/n	8355.246	22.191	1.00
	4	K8-null	4777.861	12.69	0.57

B

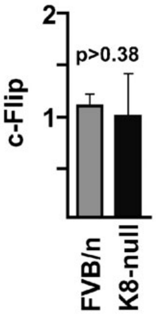

Fig. S2. c-Flip expression is similar in nontransgenic FVB/n and K8-null livers under basal conditions. Although the c-Flip band of K8-null (lane 4 in Fig. 2C) was a little bit weaker than that of FVB/n under basal conditions (lane 1 in Fig. 2C), it is likely due to the variation of c-Flip expression in the individual mouse, which is independent of K8 expression. The densitometric quantification of c-Flip expression from 3 mice/strain showed the c-Flip expression in both mice strains was similar under basal conditions. (**A**) Densitometric quantification of c-Flip expression. (**B**) Relative amount of c-Flip.
